# Notes from Australia

**Published:** 1898-02-12

**Authors:** 


### NOTES FROM AUSTRALIA.

### THE CHARITIES BILL.

A short session of Parliament terminated a few days before Christmas, and was marked by the introduction of a "Charities Bill," which was met with such a chorus of disapprobation all over the colony that it is not likely to be reintroduced, unless in a radically amended form.

The principal provisions of the Bill were: A small municipal rate, a tax on sports, enforcement of the liability of relatives for the cost of patients, and the establishment of a central council with local boards of control. The present methods of electing or appointing committees of management for the hospitals and institutions were not to be interfered with, nor was any donation that might be given to a particular institution to be diverted from its purpose.

Every hospital and other charitable institution committee, and every municipality, all over the colony met for the discussion of this Bill, which received the most sweeping condemnation ever heard of here. The boards were voted to be cumbersome and unnecessary; the proposed municipal rate was condemned as only a poor-rate under another name, as being unfair and also likely to dry up the springs of private charity; the liability of relatives was said to be too far reaching, and only the proposed tax on sport and the appropriation of a certain proportion of the licensing fees were commended. All the volunteer committees showed great reluctance to resign their duties, or to have their hospitals and institutions interfered with by Government officials.

At present the Government of Victoria apportions £100,000 per annum out of the general revenue for the maintenance of the charities—medical and benevolent. It must be confessed that the allocation of this amount is conducted on very happy-go-lucky principles, without system and without reasonableness. There is a Government inspector of charities, but he is powerless to do more than recommend. For the maintenance of the existing institutions at least £30,000 per annum more is required, and it is obvious that much economy could be effected by the amalgamation of many of the institutions, which, as with most voluntary efforts, have a tendency to overlap. In the meantime every institution is full and in debt, and for some years an average of about sixty aged and destitute persons have had to be sent to gaol for no fault but that there was no room for them elsewhere.

Some of the comments on the Bill were amusing enough. One member of a country municipality called the Ministry "wolves in sheep's clothing," and said that the Bill was simply putting one more spoke in the wheel of socialism; whilst a member of a metropolitan hospital committee complained that the regulations contained in the Bill were of a "socialistic character," inasmuch as they interfered with the domestic management of the institution—the hours of nurses and the appointment of medical officers. Another member of the same committee was alarmed at the prospect of all kinds of official busybodies sitting in judgment on the committee every time they wanted to do anything or to spend a few pounds.

Whatever be the merits of Sir George Turner's Bill, it is the first endeavour made in Victoria to systematize the charities, and it has obviously not been criticised from very lofty standpoints.

Concurrently with the agitation about the Charities Bill, the Old Age Pension Commission (which has been sitting for about three years) has issued its report. Again new taxes are proposed to support a grand scheme of pensions and homes. It is estimated that about 3,700 persons over 60 years of age would have to be provided for. It is probable that both these subjects will now retire into the background until some further advance is made with Australian federation.

